# The IPV-GBM Scale: A New Scale to Measure Intimate Partner Violence among Gay and Bisexual Men

**DOI:** 10.1371/journal.pone.0062592

**Published:** 2013-06-05

**Authors:** Rob Stephenson, Catherine Finneran

**Affiliations:** Emory University Rollins School of Public Health, Hubert Department of Global Health, Atlanta, Georgia, United States of America; McGill University Health Centre, McGill University, Canada

## Abstract

**Objectives:**

The paper describes the creation of a new scale to measure intimate partner violence (IPV) among gay and bisexual men.

**Methods:**

Seven focus group discussions were held with gay and bisexual men, focusing on defining intimate partner violence: 30 forms of IPV were identified. A venue-recruited sample of 912 gay and bisexual men was surveyed, examining definitional understanding and recent experiences of each of the 30 forms of IPV. Participants were also asked questions from the CDC definition of intimate partner violence and the short-form of the Conflicts Tactics Scale (CTS2S). Factor analysis of responses to the definitional questions was used to create the *IPV*-*GBM* scale, and the prevalence of intimate partner violence was compared with that identified by the CDC and CTS2S measures of intimate partner violence.

**Results:**

A 23-item scale, with 5 unique domains, was created, with strong internal reliability (Cronbach Alpha >.90). The *IPV*-*GBM* scale mirrored both the CDC and CTS2S definitions of intimate partner violence, but contained additional domains such as controlling violence, monitoring behaviors, emotional violence, and HIV-related violence. The new scale identified a significantly higher prevalence of IPV than either of the more commonly used measures.

**Conclusions:**

The results presented here provide encouraging evidence for a new, more accurate measure of intimate partner violence among gay and bisexual men in the U.S.

## Background

The emergence of research on intimate partner violence (IPV) among gay, bisexual, and other men who have sex with men (MSM) has demonstrated that IPV occurs in male-male partnerships at rates similar to or higher than opposite-sex partnerships [1]–[3]. Recently, researchers have documented vastly varied, though universally high, rates of IPV among MSM: between 32–78% for any form of IPV [4], [5], 12–45% for physical IPV [6], [7], and 5–33% for sexual IPV [7], [8]. Studies of IPV among gay and bisexual men have suffered from a number of methodological limitations [9], [10], including a challenge not unique to the field of same-sex IPV research: a lack of an uniform definition of IPV and use of IPV definitions non-specific to MSM [11]–[15]. Existing studies of IPV among MSM have relied upon measures of IPV that were created for use in assumedly heterosexual populations [13]. While IPV is universal across sexual orientations, it is not clear as to the extent to which the typologies of IPV – especially psychological IPV and controlling behaviors – experienced by gay and bisexual men are different to those experienced by heterosexual women, perhaps contributing to the wide range of prevalence estimates found in the literature. This paper describes the development of a new scale to measure IPV among gay and bisexual men, the *IPV-GBM* scale, contrasting the prevalence of IPV identified among gay and bisexual men using this new scale with that identified with two other commonly used measures of IPV. The new IPV-GBM scale has the potential to significantly improve the accuracy of the measurement of IPV among gay and bisexual men in the US, allowing a more accurate understanding of the relationships between IPV and health outcomes experienced by gay and bisexual men.

In studies of IPV in women, one of the most commonly used measures of IPV is the Revised Conflict Tactics Scale (R-CTS) [16], developed from the original Conflict Tactics Scale (CTS) [17], which aimed to measure the extent to which specific tactics, including acts of physical violence, were used in intimate partnerships [16]. The R-CTS updated the original CTS by including measurement of sexual coercion and consequences of physical violence (injury), and is comprised of 39 items contained in five sub-scales: physical assault, psychological aggression, negotiation, sexual coercion, and injury [16]. In 2004, Straus and Douglas updated the R-CTS and created the short-form CTS (CTS2S), a reduced, 10-item scale, including the same five sub-scales included in the R-CTS [18]. Recently, a number of studies have used the CTS2S to identify the prevalence of IPV among gay and bisexual men [5], [7], [13], [19], [20].

A number of studies have also relied on single-item questions to capture the experience of IPV among gay and bisexual men [21]–[28], often based up Center for Disease Control and Prevention (CDC)-developed specific definitions of physical and sexual IPV. Variations of this definition are commonly used to capture recent experiences of physical (*In the last “time period”, have any of your partners ever tried to hurt you? This includes pushing you, holding you down, hitting you with a fist, kicking you, attempting to strangle you, and/or attacking you with a knife, gun or other weapon?*) and sexual violence (*In the last “time period”, have any of your partners ever used physical force or verbal threats to force you to have sex when you did not want to?*) [11].

In a systematic review of the literature around IPV among MSM, Finneran and Stephenson (in press) note that across 28 studies identified, 16 different definitions of IPV were used by researchers in various combinations [13]. The most commonly used scale measures of IPV were the Conflict Tactics Scale [17] or its derivatives, the Revised Conflicts Tactics Scale [16] or the CTS2S[18]. Ten studies used definitions of IPV that were unique to the study or did not provide a reference to a validated scale, while several studies used binary measures of the presence of IPV based on the CDC definition of violence. However, none of these measures were developed specifically for gay and bisexual men; hence, it remains unknown whether or not these measures accurately represent IPV in gay and bisexual men. Several authors of studies captured in this systematic review reported having to modify the validated scales *post hoc*, such as by using gender-neutral language, in order to make the measurement tools appropriate for MSM [13]. In this paper we describe the development of a new scale – the intimate partner violence among gay and bisexual men (*IPV-GBM*) scale – and compare the prevalence of IPV identified with that identified with the CTS2S and the CDC measures of IPV.

## Methods

### Ethics

This study was approved by Emory University's Institutional Review Board.

### Data

Data collection involved two stages: the first included seven focus group discussions (FGDs) with gay and bisexual men stratified by race (Black/African-American and white) in Atlanta, Ga., and the second stage included a survey of over1000 gay and bisexual men, also in Atlanta, Ga. For both stages, respondents were recruited through venue-based sampling (VBS). VBS is a derivative of time-space sampling, in which sampling occurs within prescribed blocks of time at previously-identified venues at which hard-to-reach populations congregate with greater frequency than elsewhere [29]. In order to reach a diverse population of gay and bisexual men in the Atlanta area, the venue sampling frame used for this study consisted of a wide variety of over 160 gay-themed or gay-friendly venues, including Gay Pride events, gay sports teams events, gay fundraising events, downtown areas, gay bars, bathhouses, an AIDS service organization, an MSM-targeted drop-in center, gay bookstores, restaurants, and urban parks.

Study recruiters stood adjacent to the venue, drew an imaginary line on the ground, and approached every *n*th man who crossed it; *n* varied between one and three depending on the volume of traffic at the venue. If he agreed to be screened, he was then asked a series of eight questions to assess his eligibility. Eligible men were then read a short script that described the study process. For men recruited for the first phase, the script described their participation in a FGD. Men were eligible for FGD participation if they reported being aged 18 or older, living in the Atlanta metropolitan area, and identifying as a gay or bisexual man. In total, seven FGDs were held (n = 84); two with only African-American/Black participants, one with only white participants, and four with both participants of all races/ethnicities. Each FGD lasted approximately one hour, and the discussion centered on understanding multiple definitions of IPV. The question guide was based on the CTS2S questions [18]; respondents were asked if they would consider each item to be IPV if it were to occur in a male-male relationship. Further questions examined participant's definitions of sexual, physical, and psychological IPV and controlling/stalking behaviors. Discussions were recorded and transcribed, with analysis conducted in MAXQDA. The focus of the analysis was on identifying definitions of IPV, and on examining variations in definitions of IPV across white and Black/African-American participants. FGD transcripts were reviewed by three different researchers in order to ensure that all forms of IPV mentioned by the participants were captured, resulting in the identification 46 different potential forms of IPV. Forms of IPV that were inconsistent with the empirical literature regarding the nature of IPV (e.g., slamming a door, giving someone the “silent treatment”) were removed and duplicative forms of IPV (e.g., calling someone names and putting him down) were condensed. As a result of this review, 30 different forms of IPV were used to create the survey questions in order to examine the perceptions of and experience of IPV among the sample of over 1000 gay and bisexual men in Atlanta.

Recruitment for the survey was conducted using the same venue-based approach as was used for FGD recruitment. For survey recruitment, eligible participants were read a script that outlined their potential participation in a web-based survey, approximately 25 minutes in length, that could be completed at home, or, in the case of five venues (the AIDS service organization, the drop-in center, Atlanta Pride, In the Life Pride, and a National Coming Out Day event), at the venue itself on an iPad. Men interested in study participation were then given a card with a web address and a unique identifier, ensuring that the survey could only be completed once per venue-recruited participant.

Of 4,309 men approached, 2,936 (59.9%) agreed to be screened for the survey. Of these, 2,093 (71.3%) were eligible for study participation. Men were eligible for study participation if they reported being 18 years of age or older, being male, identifying as gay/homosexual or bisexual, living in the Atlanta Metro Area, and having had sex with a man in the previous six months. Of eligible participants, 1,965 (93.9%) were interested in study participation. A total of 1,074 men completed the survey; thus 21.9% of men approached and 51.4% of eligible men completed the survey. Approximately one-third (33.7%) completed the survey at a venue, while the remaining two-thirds (66.3%) of respondents completed the survey at home. A total of 912 men had complete data for all covariates of interest and were included in the final analysis.

The self-administered, web-based survey, hosted on SurveyGizmo, contained several domains of questions regarding demographics (e.g., age, education, and race) and recent sexual behavior with male partners. To measure IPV, the survey included 30 items taken from the FGDs: participants were asked if they considered each one of the items to be IPV (“*Would you consider it violent if a male partner of yours were to…”*), and if they had experienced it from or perpetrated it against a male partner in the past 12 months. The survey also included the CTS2S and the binary questions based on the CDC definitions measuring the experience and perpetration of physical and sexual IPV.

#### Creating a new scale to measure IPV among gay and bisexual men

Rotational factor analysis was conducted to identify which of the 30 items were to be included in the *IPV-GBM* scale based upon respondent's answers to whether or not they would consider an individual item presented to be violent if it happened to them from a male partner. The factor structure of the *IPV-GBM* scale was determined using principal components analysis with oblique rotation using a promax solution. The Kaiser-Meyer-Olkin test and Bartlett's test of sphericity were calculated prior to the exploratory factor analysis (EFA) in order to assure the appropriateness of EFA. The factor analysis was conducted for the total sample, and then separately for white and Black/African-American respondents to identify racial variations in scale content. There were insufficient numbers of Latino/Other respondents to allow factor analysis to be performed for this group. Reliability of each definitional scale (overall, white respondents, Black/African-American respondents only) was assessed by calculating Cronbach's alpha to assess the internal consistency of the items. Adequate reliability was indicated if Cronbach's alpha was >0.70.

#### The analysis sample

**Table 1** shows the characteristics of participants in the final analysis sample. The mean age was approximately 34 years, with the majority reporting post-secondary education (83.9%), current employment (79%), negative HIV status (69%), and homosexual sexual orientation (90%). Approximately 48% of the sample was white non-Hispanic, 40% Black/African-American non-Hispanic, and 12% Latino/Hispanic or other, including Asian/Pacific Islander and Native American/Alaska Native.

**Table 1:** Sample characteristics (n = 912).

Mean		Standard deviation
Age	34.5	10.6
	**%**	**n**
***Race***		
White non-Hispanic	48.0	438
Black / African-American non-Hispanic	39.3	358
Latino/Hispanic & Other	12.7	116
***Education Level***		
High School or Less	16.2	147
Some College / 2 yr. Degree	32.8	298
College or More	51.1	465
***Employment Status***		
Employed	78.9	715
Unemployed	21.1	191
***HIV Status***		
Negative	69.3	631
Positive	23.9	217
Never tested / Unknown	6.8	62
***Sexual Orientation***		
Homosexual	89.8	819
Bisexual	10.2	93
** TOTAL**	100	912

### Results

#### Definitions of IPV

There was significant variation reported among survey participants as to what constituted IPV (**Table** **2**). While more than 90% of respondents agreed that hitting, punching, kicking, rape, slapping and intentional damage to property were forms of IPV, fewer than 40% of participants reported that preventing the victim from seeing his friends or family, putting the perpetrator's sexual needs before the victim's, asking/telling the victim to act straight around others, criticizing the victim's clothes, or calling the victim fat were considered IPV. Thus, definitions of IPV tended to focus more on physical and extreme forms of sexual IPV (e.g., rape), whereas controlling behaviors were less likely to be viewed as IPV. Latino/Other men endorsed an average of 20 of 30 items as IPV and Black/African-American an average of 19 of 30, both significantly higher than the mean 17 endorsed by white men. There were racial variations in the definitions of IPV: Black/African-American participants were less likely to report that hitting, punching, kicking, rape, slapping, intentionally transmitting HIV and intentional damage to property were forms of IPV, although the vast majority of all respondents affirmed that these were forms of IPV. Conversely, Black/African-American and Latino/Other participants were more likely to report that doing something sexual for which you hadn't given consent, preventing someone from seeing their family or friends, refusing to wear a condom during sex, calling someone names, and cheating were forms of IPV. Non-white participants were also more likely to report that controlling behaviors, such as demanding access to a cell phone or email, reading text messages or email, and preventing someone from seeing his friends were forms of IPV. Whereas white men were more likely to report physical violence or extreme sexual violence as IPV, non-white men were more likely to report psychological violence or controlling behaviors as forms of IPV.

**Table 2:** Percentage of gay and bisexual men agreeing that each statement was a form of intimate partner violence.

Type of Violence	White	Black	Other	Total	p-value (<)
***Hit you***	98.6	93.3	94.8	96.1	***0.029***
***Punch you***	97.7	93.9	94.0	95.7	***0.004***
***Kick you***	97.7	93.0	96.6	95.7	***0.015***
***Rape you***	96.8	92.7	95.7	95.1	***0.000***
***Slap you***	94.7	89.7	94.8	92.8	***0.017***
Damage your property (for example, break a TV or cell phone)	94.1	90.2	93.1	92.4	0.355
Push/shove you	90.2	86.3	89.7	88.6	0.120
***Intentionally transmit HIV to you***	87.9	84.4	88.8	86.6	***0.000***
Force you to do something sexually that you didn't want to do	84.5	83.2	88.8	84.5	0.216
Lie to you about his HIV status	74.7	80.4	76.7	77.2	0.416
Not tell you he had HIV before you had sex	74.0	77.1	77.6	75.7	0.478
***Do something sexual to you for which you hadn’t given your prior consent***	68.5	75.4	78.4	72.5	***0.025***
***Prevent you from seeing your family***	60.0	64.8	73.3	63.6	***0.018***
***Refuse to wear a condom during sex***	56.8	68.4	62.1	62.1	***0.008***
***Prevent you from seeing your friends***	58.7	60.6	72.4	61.2	***0.011***
***Call you names / put you down***	54.6	63.4	66.4	59.5	***0.029***
***Cheat on you***	42.0	62.0	60.3	52.2	***0.046***
Threaten to tell someone who didn’t know you were gay/bisexual about your sexual orientation (“out you”)	44.5	53.4	57.8	49.7	0.519
***Demand access to your cell phone***	46.8	49.4	53.4	48.7	***0.005***
***Demand access to your email***	46.3	46.9	52.6	47.4	***0.000***
***Read your text messages without your knowledge***	44.1	47.8	56.9	47.1	***0.000***
***Read your email without your knowledge***	43.8	47.8	56.0	46.9	***0.000***
Unintentionally transmit HIV to you	33.6	46.6	48.3	40.6	0.263
Repeatedly post on your social networking pages (Facebook, Twitter, etc.)	33.8	46.9	45.7	40.5	0.102
***Prevent you from seeing his friends***	35.2	40.5	49.1	39.0	***0.000***
Put his sexual needs before yours	25.8	48.0	48.3	37.4	0.059
***Prevent you from seeing his family***	30.8	39.4	44.8	36.0	***0.025***
***Ask or tell you to “act straight” around certain people***	19.9	31.3	34.5	26.2	***0.004***
***Criticize your clothes***	13.5	26.3	29.3	20.5	***0.026***
***Call you fat***	17.6	21.5	25.9	20.2	***0.000***

Significant differences by race are denoted in *bold italics*.

Both the Kaiser-Meyer-Olkin test (0.903) and Bartlett's test of sphericity (χ^2^ = 17817.0, p<0.000) indicated that the variation present in the data was well-suited to EFA, both for the overall sample and for factor analysis by race (White men, KMO: 0.866, Bartlett's test p<0.000) and Black men, KMO: 0.901, Bartlett's test p <0.000). The factor analysis yielded five unique factors with eigenvalues >1.0: physical and sexual IPV, monitoring behaviors, controlling behaviors, HIV-related IPV, and emotional IPV (**Table** **3**). The same five factors were identified for each racial group, although the content of the factor varied by race. Five items did not load into any factor (i.e., alpha<0.50 in any one factor): name-calling, refusing to wear a condom during sex, revealing the victim's sexual orientation to others (“outing” him), doing something sexually for which the victim had not given his prior consent, and unintentionally transmitting HIV to the victim.

**Table 3:** Factor analysis of definitions of intimate partner violence among gay and bisexual men.

Items	Factor Loading		
	All men	White men	Black men
***Domain 1: Physical & Sexual***			
Eigenvalue (Proportion of Variance Explained)	9.6985 (0.3233)	9.21088 (0.3070)	10.20997 (0.3403)
Combined Cronbach Alpha	0.8458	0.8167	0.8987
Slap you	0.8312	0.8044	0.8836
Punch you	0.8272	0.7655	0.8756
Hit you	0.8289	0.7715	0.8769
Kick you	0.8272	0.7655	0.8775
Push you	0.8567	–	0.9021
Force you to do something sexually that you didn’t want to do	0.8717	–	0.9035
Rape you	0.8322	0.7883	0.8793
Damage your property (for example, break a TV or cell phone)	0.8458	0.8368	0.8894

*Domain 2: Monitoring*			
Eigenvalue (Proportion of Variance Explained)	4.16566 (0.1389)	3.80936 (0.1270)	4.04978 (0.1350)
Combined Cronbach Alpha	0.9226	0.9279	0.9148
Demand access to your cell phone	0.9022	0.9031	0.8997
Demand access to your email	0.8983	0.9015	0.8918
Read your text messages without your knowledge	0.8944	0.9013	0.8837
Read your email without your knowledge	0.8928	0.9002	0.8829
Repeatedly post on your social networking pages	0.9345	0.9460	0.9186

*Domain 3: Controlling*			
Eigenvalue (Proportion of Variance Explained)	1.76858 (0.0509)	1.73009 (0.0577)	1.95378 (0.0651)
Combined Cronbach Alpha	0.8860	0.8864	0.8869
Prevent you from seeing your family	0.8531	0.8522	0.8573
Prevent you from seeing his family	0.8606	0.8683	0.8541
Prevent you from seeing your friends	0.8435	0.8384	0.8452
Prevent you from seeing his friends	0.8569	0.8559	0.8618

*Domain 4: HIV-related*			
Eigenvalue (Proportion of Variance Explained)	1.47115 (0.0490)	1.56658 (0.0522)	1.44745 (0.0482)
Combined Cronbach Alpha	0.8512	0.8476	0.8326
Lie to you about his HIV status	0.7160	0.6995	0.7931
Not tell you he had HIV before you had sex	0.7156	0.6830	0.7886
Intentionally transmit HIV to you	0.8999	0.9122	0.8000
Cheat on you	–	–	0.8031
Put his sexual needs before yours	–	–	0.8349

*Domain 5: Emotional*			
Eigenvalue (Proportion of Variance Explained)	1.25644 (0.0419)	1.138720 (0.0462)	1.25642 (0.0419)
Combined Cronbach Alpha	0.7152	0.7607	0.6994
Call you fat	0.6707	0.7207	0.6422
Ask or tell you to “act straight” around certain people	0.5996	0.6990	0.5844
Criticize your clothes	0.6031	0.6898	0.5924
Put his sexual needs before yours	–	0.7092	–
**Total Chronbach Alpha for All Domains Combined**	**0.9060**	**0.8960**	**0.9147**

#### Factor One: Physical and Sexual IPV

For the total sample, and for white respondents, this factor was comprised of slapping, punching, hitting, kicking, pushing, coerced sex, rape, and damage to property: however, for white respondents, pushing and coerced sex did not load into this factor. The factor explained 32% of total variance for the total sample: 31% for white men and 34% for Black/African-American men.

#### Factor Two: Monitoring Behaviors

The same items loaded for all groups: demanding access to a cell phone, demanding access to email, reading text messages or email(s) without knowledge, and repeatedly posting on victim's social networking pages (e.g., Facebook, Twitter), explaining approximately 14% of total variance.

#### Factor Three: Controlling Behaviors

Again, the same items loaded for all groups: preventing a victim from seeing his family or friends, and preventing victim from seeing his partner's family or friends, explaining approximately 5% of the variance in each group.

#### Factor Four: HIV-related IPV

For the total sample and for white men, the items loading in this factor were lying about HIV status to a partner, not revealing HIV positive status to a partner before sex, and intentionally transmitting HIV, which collectively explained 5% of the total variance. For Black/African-American men, cheating and the perpetrator putting his sexual needs first also loaded on this factor, although the percentage of variation explained remained approximately 5%.

#### Factor Five: Emotional IPV

For the total sample and for Black/African-American men, the following items loaded: calling the victim fat, asking/telling the victim to “act straight,” and criticizing the victim's clothes, explaining approximately 4% of the variation. For white men, the perpetrator putting his sexual needs before the victim's also loaded on this factor. This factor explained only 4% of the total variance.

#### The experience of IPV

Among the total sample, the most commonly experienced forms of IPV in the past 12 months were criticizing of clothes (18.5%, emotional IPV), reading text messages without permission (16.2%, monitoring behavior), and pushing/shoving (14.7%, physical and sexual IPV). The least commonly experienced forms of IPV were rape (2.8%, physical and sexual IPV), preventing victim from seeing his family (3.3%, monitoring behaviors), and intentionally transmitting HIV (3.2%, HIV-related IPV) **(** **Table** **4**).

**Table 4:** Percentage of gay and bisexual men reporting the experience and perpetration of each form of intimate partner violence and results of chi-square testing.

	Receipt of IPV					Perpetration of IPV				
Type of Violence	White	Black	Other	Total	p-value (<)	White	Black	Other	Total	p-value (<)
***Domain 1: Phy1sical & Sexual***										
Punch/Hit/Slap you	5.9	14.5	12.1	10.0	***0.000***	2.8	9.8	6.3	6.1	***0.000***
Kick you	3.0	9.3	6.0	5.8	***0.001***	1.3	6.7	5.1	4.0	***0.000***
Push/shove you	8.8	20.9	18.1	14.7	***0.000***	6.9	13.3	13.9	10.3	***0.005***
Force you to do something sexually that you didn’t want to do	5.3	8.7	7.8	6.9	0.161	1.9	6.1	6.3	4.1	***0.004***
Rape you	2.1	3.9	1.8	2.8	0.212	1.3	4.4	3.8	2.8	***0.018***
Damage your property (for example, break a TV or cell phone)	4.8	12.4	10.4	8.5	0.001	1.9	8.6	5.1	5.0	***0.000***
** Any Physical/Sexual IPV**	**15.1**	**30.6**	**25.3**	**22.6**	**0.000**	**12.9**	**26.7**	**24.1**	**19.7**	***0.000***

*Domain 2: Monitoring*										
Demand access to your cell phone	4.4	16.0	15.6	10.4	***0.000***	3.6	13.0	8.9	8.1	***0.000***
Demand access to your email	2.3	10.6	8.7	6.4	***0.000***	1.9	8.3	3.8	4.8	***0.000***
Read your text messages without your knowledge	13.1	18.8	20.2	16.2	***0.045***	13.1	15.4	7.6	13.2	0.109
Read your email without your knowledge	7.8	14.3	15.7	11.4	***0.005***	8.1	12.3	2.5	9.4	***0.010***
Repeatedly post on your social networking pages	6.8	12.0	6.3	8.8	***0.024***	3.0	8.1	5.1	5.4	***0.004***
** Any Monitoring IPV**	**18.5**	**26.2**	**31.7**	**22.9**	***0.004***	**16.2**	**19.6**	**13.9**	**17.4**	0.289

*Domain 3: Controlling*										
Prevent you from seeing your family	1.6	5.6	2.6	3.3	***0.007***	2.6	4.9	3.8	3.7	0.187
Prevent you from seeing his family	5.3	6.4	6.1	5.8	0.774	0.4	5.4	2.5	2.7	***0.000***
Prevent you from seeing your friends	4.4	8.2	7.8	6.2	0.068	1.9	5.6	5.1	3.8	***0.012***
Prevent you from seeing his friends	5.3	6.7	7.8	6.2	0.503	0.6	5.6	3.8	3.0	***0.000***
** Any Controlling Behaviors**	**9.4**	**13.7**	**19.0**	**12.0**	**0.020**	**3.4**	**8.1**	**8.9**	**5.9**	***0.007***

*Domain 4: HIV-related*										
Lie to you about his HIV status	3.7	12.4	2.6	6.9	***0.000***	1.1	7.1	3.6	3.9	***0.000***
Not tell you he had HIV before you had sex	2.8	12.6	7.8	7.3	***0.000***	1.9	9.3	3.9	5.2	***0.000***
Intentionally transmit HIV to you	0.7	6.5	2.6	3.2	***0.000***	0.4	4.4	2.5	2.3	***0.000***
** Any HIV-Related IPV**	**4.0**	**18.3**	**8.9**	**10.5**	***0.000***	**2.1**	**10.8**	**6.3**	**6.2**	***0.000***

*Domain 5: Emotional*										
Call you fat or ugly	13.0	12.0	16.4	13.0	0.484	4.9	8.1	11.4	6.8	***0.041***
Ask or tell you to “act straight” around certain people	6.9	10.9	11.3	9.0	0.093	3.2	6.2	5.1	4.6	0.114
Criticize your clothes	21.2	15.1	18.3	18.5	0.087	14.1	13.7	11.4	13.7	0.814
** Any Emotional IPV**	**30.0**	**28.4**	**30.4**	**29.3**	0.848	**16.8**	**19.8**	**17.7**	**18.2**	0.514
** Any ** ***IPV-GBM*** ** IPV**	**44.7**	**51.1**	**49.4**	**47.8**	0.158	**32.6**	**40.8**	**40.5**	**36.7**	***0.031***

Significant differences are denoted in ***bold italics***.

There were clear racial variations in reporting experience of IPV. Black/African-American respondents were more likely to report experiencing any form of physical and sexual IPV and all forms of HIV-related IPV. Black/African-American respondents were also more likely to report being prevented from seeing their family by a male partner, but were not more likely than white men to report any other controlling behaviors. There were no significant racial differences in the reporting of emotional violence between Black/African-American and white men. Similar variations were present in the variation of reporting recent perpetration of IPV, with Black/African-American men significantly more likely to report any recent perpetration of IPV.

**Figure 1:**
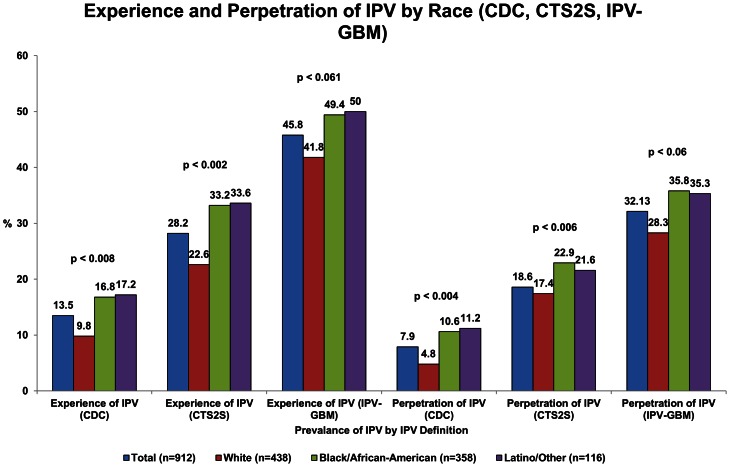
Receipt of IPV and Perpetration of IPV, in total and by race, as measured by the CTS2S, CDC, and IPV-GBM definitions of IPV, and chi-square p-values.

The CDC definition-based measure of IPV consistently generated the lowest prevalence of IPV (experience 13.5%, perpetration 7.9%). The CTS2S measure generated higher prevalences (experience 28.2%, perpetration 18.6%), while the newly developed *IPV-GBM* scale generated significantly higher prevalences than the other two measures (experience 45.8%, perpetration 32.1%). The same patterns in the reporting of IPV experience and perpetration across the three measures were observed in each racial group; however, while the CDC measurement and the CTS2S measurement suggested significantly higher prevalences of both experience of IPV and perpetration of IPV among Black/African-American and Latino/Other men compared to white men, these differences were not statistically significant when using the IPV-GBM scale (**Figure 1**).

The results of sensitivity analysis are summarized in **Table** **5**. For all but two forms of IPV (rape and forced sex), the CTS2S measurement was more sensitive at identifying victims of IPV compared to the CDC measurement. For example, only 27% of participants who had recently experienced emotional violence from a male partner were classified as experiencing violence by the CDC measurement, whereas the CTS2S correctly classified 66% of these participants. Overall, the 64.9% and 41.7% of participants with recent experience of IPV per the IPV-GBM scale were not classified as such by the CDC measurement and the CTS2S measurement, respectively.

**Table 5:** Results of sensitivity analysis: comparisons are made between participants reporting experience of IPV based on the *IPV*-*GBM* items and domains versus both the CDC and CTS2S definitions of IPV.

	n	% Answering yes to CDC (physical or sexual)	% Answering yes to any CTS2S question
***Domain 1: Physical & Sexual***			
Punch/Hit/Slap you	92	57.4	100.0
Kick you	53	48.3	100.0
Push/shove you	133	56.3	100.0
Force you to do something sexually that you didn’t want to do	63	39.1	37.7
Rape you	60	30.3	30.3
Damage your property (for example, break a TV or cell phone)	132	51.1	85.6
** Any Physical/Sexual**	**224**	**47.2**	**86.1**

*Domain 2: Monitoring*			
Demand access to your cell phone	94	45.7	73.3
Demand access to your email	58	54.3	80.0
Read your text messages without your knowledge	147	39.7	65.5
Read your email without your knowledge	103	36.6	64.0
Repeatedly post on your social networking pages	78	46.5	66.3
** Any Monitoring**	**200**	**35.6**	**60.3**

*Domain 3: Controlling*			
Prevent you from seeing your family	30	40.0	75.0
Prevent you from seeing his family	53	43.6	62.9
Prevent you from seeing your friends	57	40.0	75.4
Prevent you from seeing his friends	57	40.6	70.3
** Any Controlling**	**106**	**39.1**	**66.1**

*Domain 4: HIV-related*			
Lie to you about his HIV status	63	32.4	64.9
Not tell you he had HIV before you had sex	66	35.1	58.1
Intentionally transmit HIV to you	29	31.4	65.7
** Any HIV-Related**	**92**	**39.6**	**63.4**

*Domain 5: Emotional*			
Call you fat or ugly	119	33.6	100.0
Ask or tell you to “act straight” around certain people	82	34.8	67.4
Criticize your clothes	168	27.1	60.8
** Any Emotional**	**263**	**27.4**	**66.2**
** Any ** ***IPV-GBM*** ** IPV**	**418**	**35.1**	**58.3**

NB: n varies by row.

## Discussion

The results demonstrate the increased sensitivity of scale to specifically measure the experience of IPV among MSM in the US. The *IPV-GBM* scale, consisting of 23 items in five unique domains of IPV, showed strong internal reliability, and more than 60% of variance in definitions of IPV was explained by the scale items. Although there were some variations in the content of the scale by race, these were minimal, and the scale seems appropriate for use in both white and Black/African-American gay and bisexual male populations. A small sample size limited the ability to create the scale for Latino or other racial/ethnic groups, and further work must examine the extent to which the *IPV-GBM* scale is applicable to other racial and ethnic groups of gay and bisexual men in the U.S Further work is required to test associations between IPV as measured in the *IPV-GBM* and other health outcomes, especially health outcomes thought to have strong correlations with IPV such as substance abuse [4], [30]–[34], depression [4], [5], [32], and HIV risk. Much has been written recently about the possible links between IPV and HIV among gay and bisexual men in the US [8], [9], [13], [19], [27], [32], [35], although findings of a statistical association have been mixed [8], [19], [28], [34], [36], [37].

Participants largely conceptualized IPV as including physical violence and extreme sexual coercion, items that are included in both the CDC definition of IPV and the variations of the CTS. However, the *IPV-GBM* also identified areas of IPV not included in other measures that gay and bisexual men reported as constituting IPV. These included HIV-related IPV, monitoring behaviors (such as observing emails/texts) and controlling behaviors (including limiting access to friends or family). The *IPV-GBM* identified a significantly higher prevalence of IPV than either of the other two measures tested, suggesting that the inclusion of items in an IPV scale that more closely reflects the lived experiences of gay and bisexual men may lead to a more accurate, although higher, estimation of the prevalence of IPV. Of course, it is also possible that these additional forms of IPV are also prevalent in heterosexual populations, and further work is required to establish whether the newly created scale is unique to gay and bisexual men, or represents a generally more accurate measure of IPV that can be used in wider populations.

Interestingly, Black/African-American men were less likely to report that physical acts constituted IPV while they were more likely to report controlling behaviors as IPV. However, Black/African-American men were more likely to report recent experience of all forms of physical and sexual IPV, leading to a generally higher prevalence of IPV among Black/African-American gay and bisexual men. Disconnect exists between what Black/African-American men in this sample think of as IPV and what they report actually experiencing, suggesting that physical and sexual IPV were perhaps more tolerated (and hence not conceived of as IPV as often) among Black/African-American men in this sample. Despite this, the majority of men in the sample endorsed physical and sexual violence as forms of IPV. Thus, further work is needed to understand the factors driving the racial variations observed in both the conceptualization of and the experience of IPV.

There are a number of limitations to the current study. The sampling procedures relied on venue-based sampling rather than random sampling: however, there is increasing evidence that this form of sampling produces a sample of similar diversity as is found with random sampling methods [29], [38], [39]. The data are specific to the metro-Atlanta area, and there may be regional differences in how gay and bisexual men experience and conceptualize IPV; thus, further work is needed to replicate this work with men in other parts of the U.S. Additionally, the stem question used to determine whether or not something was IPV (“Would you consider it violent if a male partner of yours were to…”) was intentionally oblique. Participants may have considered that certain acts would not always necessarily constitute violence (particularly in cases of consensual violence, such as occurs in bondage, domination, and sadomasochism [BDSM] relationships); this may partly explain why no single IPV item received 100% endorsement.

## Conclusion

The results presented here provide encouraging evidence for a new, more accurate, measure of IPV among gay and bisexual men in the U.S. The large number of items in the scale enhances content sensitivity and reliability, and provides the ability to differentiate between five domains of IPV. The IPV-GBM utilizes interspersed item order to limit response set bias [40], and the referent time period can be adjusted from 12 months to meet the needs of the research question (e.g., last 3 months). The *IPV-GBM* requires a 6^th^ grade reading level, and takes approximately 10–15 minutes to complete. These characteristics are similar to those of the variations of the CTS [16], [18], which have gained in popularity and frequency of use in the IPV research community. Given the increased attention to IPV among gay and bisexual men, a more accurate measure of IPV that is grounded in the lived realities of gay and bisexual men is vital. Further work is now required to test this scale on larger samples of gay and bisexual men, and to explore the extent to which the *IPV-GBM* scale is applicable to other racial/ethnic groups and is associated with other health outcomes.
